# Survey of Thai Physicians’ Practice in Pediatric Septic Shock

**DOI:** 10.3390/children11050597

**Published:** 2024-05-15

**Authors:** Sirapoom Niamsanit, Teerapat Saengthongpitag, Rattapon Uppala, Phanthila Sitthikarnkha, Leelawadee Techasatian, Suchaorn Saengnipanthkul

**Affiliations:** 1 Department of Pediatrics, Faculty of Medicine, Khon Kaen University, 123 Mittraphap Road, Muang, Khon Kaen 40002, Thailand; sirani@kku.ac.th (S.N.); puntsi@kku.ac.th (P.S.); leelawadee@kku.ac.th (L.T.); suchsa@kku.ac.th (S.S.); 2 Department of Pediatrics, Faculty of Medicine, Suranaree University of Technology, Nakhon Ratchasima 30000, Thailand; teerapatsa@kkumail.ac.th

**Keywords:** sepsis, management, survey, shock, children

## Abstract

(1) Background: Sepsis management in children is crucial, especially in emergency services. This study aims to evaluate Thai physicians’ knowledge gaps in the emergency management of sepsis in children and to evaluate their adherence to the current sepsis clinical practice guidelines. (2) Methods: This is a cross-sectional survey of Thai physicians’ management of septic shock in children. The survey was conducted through online questionnaires from March 2019–April 2019. (3) Results: Of the 366 responders, 362 (98.9%) were completed. Most of the responders were general practitioners (89.2%) and pediatricians (10.8%). The time from positive sepsis screening to being evaluated by physicians within 15 min was reported by 83.9%. The most common choice of fluid resuscitation was normal saline solution (77.3%). The practice of a fluid loading dose (20 mL/kg) consistent with the guidelines was 56.3%. The selection of the first vasoactive agent in warm shock (norepinephrine) and cold shock (epinephrine) according to recommendations in the guidelines was 74.3% and 36.2%, respectively. There was a significant difference between general practitioners and pediatricians in terms of knowledge about initial fluid resuscitation and the optimal vasoactive agent in cold shock (*p-*value < 0.001). In the multivariate model, factors associated with the guideline-based decision-making of vasoactive agent choice for cold shock were specialist training (pediatrician) and the completion of sepsis management training certification, with adjusted odds ratios (AORs) of 7.81 and 2.96, but working experience greater than ten years was inconsistent with the guideline-based decision-making (AOR 0.14). (4) Conclusions: Thai clinicians were unfamiliar with pediatric sepsis therapy standards, specifically the quantity of early fluid resuscitation and the appropriate vasoactive medications for cold shock. To encourage adherence to the guidelines, we propose a regularly required training course on pediatric sepsis management.

## 1. Background

Sepsis is a common acute illness that can potentially result in death and can be found in all hospitals [1]. It is also one of the leading causes of death in children, with a mortality rate ranging from 4% to 50% globally [1,2,3,4,5]. Recent multicenter research in an Asian community revealed that the mortality rate for septic shock in children was 19.2%. Before the Surviving Sepsis campaign guidelines were implemented in Thailand, the mortality rate for septic shock among children ranged from 37 to 42 percent [6]. The annual death rate ranged from 231 to 522 per 100,000 persons [7,8].

In 2020, the Surviving Sepsis Campaign International Guidelines for the Management of Septic Shock and Sepsis-associated Organ Dysfunction in Children were introduced, offering clinicians critical guidance for treating children with septic shock [9]. The use of a protocolized sepsis guideline was associated with improvement in organ dysfunction and reduced the overall fatality rate [10,11]. Despite this, there is a notable gap in guideline adherence, particularly in emergency room settings. Moreover, mortality rates still vary significantly across the country, reflecting inconsistencies in the application of these guidelines.

Thailand developed clinical practice guidelines for the management of pediatric sepsis in 2018 [12]. Tertiary care centers that adhere to the sepsis protocol or the sepsis bundle recommendations have shown a substantial reduction in mortality, ranging from 18 to 42 percent [10]. Nonetheless, while explicit sepsis protocols are associated with reduced mortality, factors such as regional healthcare infrastructure, availability of intensive care, and public awareness also critically impact early recognition and treatment of sepsis, thereby influencing survival rates [13]. The majority of pediatric sepsis-related deaths occur within 48 h of the onset of symptoms [10]. Inaccurate estimates of pediatric sepsis are hampered by errors in diagnostic coding, which leads to severe underreporting; concurrently, a higher awareness of sepsis may contribute to earlier discovery of the condition, resulting in an increase in apparent survival rates [14].

The estimated population of our nation is 66 million, with over 13 million children. Due to the limited resources available at the primary hospital, emergency care in our nation is administered by general physicians rather than pediatricians, resulting in a wider range of treatment options and a lower incidence of uniformity. The ratio of general practitioners to pediatricians in Thailand is approximately 15:1 per year. Furthermore, Thai physicians have limited national survey data regarding the practical management of sepsis resuscitation in pediatric patients. Therefore, we designed a web-based survey to evaluate pediatric sepsis guideline adherence in this common emergency illness.

## 2. Methods

The questionnaires were designed to evaluate knowledge (File S1) regarding sepsis management in children according to the clinical practice guidelines of Thailand’s Society of Pediatric Pulmonology and Critical Care. Questionnaire content validity was tested by validity index for individual items (item content validity index) methods by pulmonologists and intensivists, using a 4-point Likert scale: 1 = not relevant, 2 = somewhat involved, 3 = involved, 4 = strongly involved. The items that were scored 3–4 by everyone were used in the test. The questionnaire was tested for reliability by convenience sampling, using 20 general practitioners who obtained Cronbach’s alpha from the pilot test = 0.721. The questionnaire consisted of 3 parts: general information about responders, current practice regarding the treatment of sepsis in children, and additional suggestions regarding sepsis management. The online web-based self-administered questionnaire was open and publicly distributed from March 2019 to April 2019. Participation in the study was voluntary. All physicians agreeing to participate signed an online consent form before proceeding to the questionnaire. All data collected were confidential.

Demographic data were analyzed using descriptive statistics and presented as percentages, means, and standard deviations. If the distribution of these data did not conform to a normal distribution, medians and interquartile ranges were used instead. The different practices between general practitioners and pediatricians were evaluated using regression analysis with logistic transformation. A *p*-value < 0.05 was considered to indicate statistically significant differences. Adjusted odds ratios (ORs) and their 95% confidence intervals (CIs) were reported to denote the strength of the association. All data analysis was carried out using STATA version 10.0 (StataCorp, College Station, TX, USA).

Online nonverbal informed consent was obtained before starting the questionnaire. The research objectives were given to the responders, ensuring that there was no impact on the subject. This study was approved by the institutional review board of Khon Kaen University, Human Research Ethics Committee (#HE611453).

## 3. Results

During the study period, a total of 362 out of 366 (98.9%) responses were completed by all physicians, divided into 323 general practitioners (89.2%) and 39 pediatricians (10.8%).

There were 166 (45.9%) males and 196 (54.1%) females. Most participants had working experience of 2–3 years (37.3%). The survey responders’ demographic details are shown in Table 1.

Number of pediatric patients with sepsis and general knowledge regarding the principle of treatment.

Most physicians treated pediatric patients with sepsis 1–2 cases per month (63.7%), followed by 2–5 cases (19.2%). The time to see the doctor after the screening process for pediatric septic shock within 5 min and 15 min was 43.5% and 40.4%, respectively.

Most physicians thought that the most important aspect for resuscitation of pediatric septic shock was fluid management (80.9%), followed by broad-spectrum antibiotics (19.2%); most of the responders prescribed antibiotics within 1 h (97.0%).

### 3.1. Fluid for Resuscitation

Most physicians (77.4%) used normal saline as the first choice for fluid resuscitation, followed by Ringer’s lactate (16.0%) and 5% dextrose in normal saline (5.8%). The accurate loading dose (20 mL/kg) was answered in 56.4% of cases, followed by 10 mL/kg (35.4%) and 30 mL/kg (6.6%) (Table 2).

### 3.2. Vasoactive Agents

The availabilities of each vasoactive agent were as follows: epinephrine 93.6%, dopamine 92.5%, norepinephrine 85.4%, dobutamine 54.9%, vasopressin 11.3%, terlipressin 5.9%, levosimendan 3.9%, and angiotensin 1.4%. When physicians ordered vasoactive agents, 29.3% of nurses administered the drug within 5 min, 38.4% within 15 min, 21.8% within 30 min, and 9.9% more than 30 min.

For warm shock, 74.3% of physicians chose norepinephrine as the first choice for a vasoactive agent, consistent with the guidelines. The others chose dopamine (11.6%), followed by epinephrine (10.5%). For cold shock, 36.2% of physicians chose epinephrine as the first choice for a vasoactive agent, consistent with the guidelines, followed by norepinephrine (32.3%), dopamine (21.6%), and dobutamine (5.8%) (Table 2).

### 3.3. Training of Sepsis Management in Children

A total of 58.0% of the responders had never attended a formal training course on pediatric sepsis management. Moreover, 98.0% of the responders agreed with the yearly mandatory review of pediatric sepsis guidelines.

### 3.4. Central Venous Catheterization

A total of 85.1% of physicians could not perform central venous catheter insertion procedures. Most of them are general practitioners; neither 48.7% of pediatricians could do so.

### 3.5. The Difference between General Practitioners and Pediatricians and Factors Associated with Guideline-Based Decision-Making

The number of physicians who chose the initial fluid loading dose correctly as recommended in the guidelines was 52.3% (general practitioners) and 89.7% (pediatricians) (*p* < 0.001). The number of physicians who answered agreeing with the guidelines for initial vasoactive agents in cold shock was 104 (32.2%) in the general practitioner group and 27 (69.2%) in the pediatrician group (*p* < 0.001) (Table 3). In the multivariate model, factors associated with guideline-based decision-making on choosing a vasoactive agent in cold shock were specialist training (general pediatrics), completion of sepsis management training certification, and working experience greater than ten years with adjusted odds ratios (AORs) of 7.81, 2.96, and 0.14, respectively. Working experience greater than ten years was associated with incorrect selection of vasoactive agents in cold shock (AOR 0.14) (Table 4).

### 3.6. Suggestions from the Responders

Some hospitals did not have local guidelines for pediatric sepsis. Therefore, some physicians did not correctly administer fluid therapy and did not determine which vasoactive agents were available at their hospitals. Most of the responders suggested regular training and review for sepsis in children in each hospital every year.

## 4. Discussion

The results of this survey confirm that overall, physicians’ knowledge regarding sepsis management in children was limited, especially regarding vasoactive agent selection and fluid therapy. The crucial point of this study is to identify the gaps of knowledge among general practitioners and subspecialties in developing countries to increase the survival rate in children with septic shock. Septic shock accounted for 20% of the pediatric intensive care unit (PICU) admission rate. The mortality rate is approximately 20–50%. Factors influencing mortality include the degree of fluid overload, pre-existing health conditions, and multiorgan failure [15,16,17,18,19]. If physicians followed the sepsis guidelines, the mortality rate could be reduced by approximately 20% [10]. In our study, the knowledge regarding guidelines did not correlate with longer working experience among general practitioners, highlighting the necessity for comprehensive training and clear protocols for managing pediatric septic shock, particularly in countries with limited pediatrician availability.

According to the Thai clinical practice guidelines for the management of pediatric sepsis and septic shock, which are based on the 2017 guidelines from the American College of Critical Care Medicine, physicians are mandated to assess patients within 15 min of suspected sepsis [20,21]. The questionnaire from this research found that 83% were consistent with guidelines’ recommendations, but 17% still took more than 15 min. The guidelines recommend administering 20 mL/kg per bolus of fluid. Nevertheless, this research found that only 56.4% of physicians gave fluid resuscitation consistent with the guidelines, of which 52.3% were general practitioners and 89.7% were pediatricians.

Previous studies have found that improper fluid resuscitation in the first hour resulted in hypovolemia, causing an increase in the mortality rate [22]. Therefore, this accentuates the need for training. Fluid management for patients with sepsis should be a mandatory skill for all physicians working in emergency care [23].

Most physicians preferred norepinephrine (74.3%) as the first choice, corresponding to the guidelines for fluid refractory shock and warm shock [9]. For cold shock, epinephrine is recommended as the first-line vasoactive agent [21,24], with correct inotrope selection being crucial as it can significantly reduce mortality rates as per the Surviving Sepsis Campaign International Guidelines [9]. However, in our study, only 32.2% of the general practitioners and 69.2% of the pediatricians used epinephrine as the first drug. Most physicians may be familiar with the adult septic shock guideline, which suggests norepinephrine as the first choice [25].

According to physicians, the main reason that the current management of pediatric sepsis was not consistent with the guidelines was that many responders (58.0%) had never received training on sepsis management in children. Additionally, the lack of availability of suitable inotropic drugs in some hospitals was a significant barrier. Therefore, sepsis guidelines should be prepared at all hospitals in their unique setting when the availability of medications is limited.

The Thai septic shock guidelines highlight the importance of early recognition and initial stabilization, including oxygen provision, fluid resuscitation, empirical antimicrobial therapy, and vasoactive medications within the first hour, while considering the varying capabilities of hospitals [21]. However, the recent study by Choudhary R et al. underlines the problem of septic shock underdiagnosis in rural hospitals, where general practitioners admit only 2% of sepsis-diagnosed children to the ICU [26]. Mortality from septic shock is high in regions where diagnosis is delayed, treatment is late, and adherence to guidelines is poor [13,27]. Consequently, regular and annual training in the management of pediatric septic shock should be implemented at every hospital level to increase compliance with treatment guidelines and enhance future survival rates.

Overall, our study showed the gaps in the knowledge regarding the guidelines and encouraged the awareness of all physicians about the necessity of compulsory courses. Moreover, regular training should be provided; a recent study documents a decline in the clinical performance resuscitation course after eight months of training [28]. Our data suggest that educational programs should be provided to all physicians to improve adherence to the updated guidelines, especially in emergency care.

Our study had certain limitations regarding response bias since self-reporting might have a false interpretation of the questionnaire or poor recognition of clinical practice. The majority of respondents were from the northeastern region due to the distribution launched online from this region. It is unclear if the respondents are overall representative of all surveyed populations. The number of general practitioners and pediatricians is significant because the proportion of specialists in Thailand is still vastly different.

## 5. Conclusions

Thai physicians have limited knowledge regarding sepsis management in children, especially the amount of initial fluid resuscitation and the appropriate vasoactive agents in cold shock. We suggest a compulsory training course on pediatric sepsis management to improve guideline adherence, particularly in general practice, which is the mainstay of this emergency condition. Furthermore, we suggest developing sepsis guidelines in each hospital to increase the survival rate, especially in a country with limited resources.

## Figures and Tables

**Table 1:** Baseline characteristics.

Participant Characteristics	Number of Participants (%) (N = 362)
Gender	
Male	166 (45.9)
Female	196 (54.1)
Working experience (years)	
0–1	75 (20.7)
1–2	48 (13.3)
2–3	135 (37.3)
3–5	55 (15.2)
5–10	29 (8.0)
>10	20 (5.5)
Medical specialist	
General practitioner	323 (89.2)
Pediatrician	39 (10.8)
Training course in sepsis/septic shock	
Yes	152 (42.0)
No	210 (58.0)
Type of hospital	
Primary care center (≤30 beds)	72 (19.9)
Secondary care center (>30 beds)	85 (23.5)
Tertiary care center	91 (25.1)
Super tertiary care	100 (27.6)
Private hospital	14 (3.9)
Region	
North	23 (6.4)
Northeast	220 (60.8)
Eastern	18 (5.0)
Central	31 (8.6)
Bangkok	28 (7.7)
Western	4 (1.1)
South	38 (10.5)

**Table 2:** Characteristics of guideline-based decision-making.

Treatment Bundle	General Practitioners (%) N = 323	Pediatricians (%) N = 39	Total (%) N = 362
The first-choice fluid resuscitation			
Normal saline *	254 (78.6)	26 (66.7)	280 (77.4)
Ringer’s lactate *	45 (13.9)	13 (33.3)	58 (16.0)
5% Dextrose in normal saline	21 (6.5)	0 (0)	21 (5.8)
Not defined	3 (0.9)	0 (0)	3 (0.8)
Amount of initial fluid given per dose			
10 mL/kg	124 (38.4)	4 (10.3)	128 (35.4)
20 mL/kg *	169 (52.3)	35 (89.7)	204 (56.4)
25 mL/kg	2 (0.6)	0 (0)	2 (0.6)
30 mL/kg	24 (7.4)	0 (0)	24 (6.6)
Not defined	4 (1.2)	0 (0)	4 (1.1)
The first-choice vasoactive agents in warm shock			
Dopamine	37 (11.5)	5 (12.8)	42 (11.6)
Dobutamine	6 (1.9)	1 (2.6)	7 (1.9)
Epinephrine	33 (10.2)	5 (12.8)	38 (10.5)
Norepinephrine *	241 (74.6)	28 (71.8)	269 (74.3)
Not defined	6 (1.9)	0 (0)	6 (1.7)
The first-choice vasoactive agents in cold shock			
Dopamine	70 (21.7)	8 (20.5)	78 (21.6)
Dobutamine	20 (6.2)	1 (2.6)	21 (5.8)
Epinephrine *	104 (32.2)	27 (69.2)	131 (36.2)
Norepinephrine	115 (35.6)	2 (5.1)	117 (32.3)
Milrinone	6 (1.7)	0 (0)	6 (1.7)
Not defined	8 (2.5)	1 (2.6)	9 (2.5)

* Consistent with the guidelines.

**Table 3:** The different practices between general practitioners and pediatricians.

Question	General Practitioners (%)	Pediatricians (%)	χ^2^	*p* Value
The first-choice fluid resuscitation (N = 362)				
Correct	299 (92.6)	39 (100)	-	0.092 #
Incorrect	24 (7.4)	0 (0)		
Amount of initial fluid given per dose (N = 362)				
Correct	169 (52.3)	35 (89.7)	19.81	<0.001 *
Incorrect	154 (47.7)	4 (10.3)		
The first-choice vasoactive agents in warm shock (N = 362)				
Correct	278 (86.1)	33 (84.6)	0.06	0.805
Incorrect	45 (13.9)	6 (15.4)		
The first-choice vasoactive agents in cold shock (N = 362)				
Correct	104 (32.2)	27 (69.2)	20.66	<0.001 *
Incorrect	219 (67.8)	12 (30.8)		

* *p-*value < 0.05. # Fisher’s exact test.

**Table 4:** Factors associated with guideline-based decision-making regarding vasoactive agents in cold shock using multiple logistic regression.

Factors	Crude Odds Ratio (95%CI)	Adjusted Odds Ratio (95%CI)	*p-*Value
Working experience			
0–1 year	1	1	
1–2 years	1.24 (0.57–2.67)	1.31 (0.59–2.89)	0.510
2–3 years	0.85 (0.46–1.59)	0.93 (0.49–1.77)	0.831
3–5 years	0.06 (0.98–4.17)	1.52 (0.70–3.31)	0.286
5–10 years	5.02 (1.99–12.70)	1.76 (0.58–5.36)	0.321
>10 years	1.51 (0.54–4.18)	0.14 (0.02–0.97)	0.047 *
Medical specialist			
General practitioner	1	1	
Pediatrician	4.74 (2.31–9.72)	7.81 (1.55–39.24)	0.013 *
Training course in sepsis/septic shock			
No	1	1	
Yes	3.85 (2.45–6.05)	2.96 (1.82–4.79)	<0.001 *

* *p*-value < 0.05.

## Data Availability

The datasets generated and/or analyzed during the current study are not publicly available due to privacy reason but are available from the corresponding author (R.U.) on request.

## Supplementary Materials

The following supporting information can be downloaded at: https://www.mdpi.com/article/10.3390/children11050597/s1, File S1: Participant Questionnaire: Survey on the Management of Pediatric Sepsis and Septic Shock among Thai Physicians.

## Abbreviations

AOR: adjusted odds ratios; AUC: CI: confidence interval; PICU: pediatric intensive care unit.

## References

[1] Ames S.G., Davis B.S., Angus D.C., Carcillo J.A., Kahn J.M. (2018). Hospital Variation in Risk-Adjusted Pediatric Sepsis Mortality. Pediatr. Crit. Care Med..

[2] Balamuth F., Weiss S.L., Neuman M.I., Scott H., Brady P.W.M., Paul R., Farris R.W.D., McClead R., Hayes K.B., Gaieski D. (2014). Pediatric severe sepsis in U.S. children’s hospitals. Pediatr. Crit. Care Med..

[3] Evans I.V.R., Phillips G.S., Alpern E.R., Angus D.C., Friedrich M.E., Kissoon N., Lemeshow S., Levy M.M., Parker M.M., Terry K.M. (2018). Association Between the New York Sepsis Care Mandate and In-Hospital Mortality for Pediatric Sepsis. JAMA.

[4] Odetola F.O., Gebremariam A., Freed G.L. (2007). Patient and hospital correlates of clinical outcomes and resource utilization in severe pediatric sepsis. Pediatrics.

[5] Prout A.J., Talisa V.B., Carcillo J.A., Mayr F.B., Angus D.C., Seymour C.W., Chang C.-C.H., Yende S. (2018). Children with Chronic Disease Bear the Highest Burden of Pediatric Sepsis. J. Pediatr..

[6] Samransamruajkit R., Wong J.J.-M., Smathakane C., Anantasit N., Sunkonkit K., Ong J., Lee O.P.E., Lee P.-C., Phumeetham S., Sultana R. (2021). Pediatric Severe Sepsis and Shock in Three Asian Countries: A Retrospective Study of Outcomes in Nine PICUs. Pediatr. Crit. Care Med..

[7] Strategy and Planning Division, Ministry of Public Health (2017). Public Health Statistics A.D. https://spd.moph.go.th/wp-content/uploads/2022/11/Hstatistic60.pdf.

[8] Sutra S., Chirawatkul A., Leelapanmetha P., Sirisuwan S., Thepsuthammarat K. (2012). Evaluation of causes-of-death: Which statistics should we rely on, hospital deaths or vital statistics?. J. Med. Assoc. Thai..

[9] Weiss S.L., Peters M.J., Alhazzani W., Agus M.S.D., Flori H.R., Inwald D.P., Nadel S., Schlapbach L.J., Tasker R.C., Argent A.C. (2020). Surviving sepsis campaign international guidelines for the management of septic shock and sepsis-associated organ dysfunction in children. Intensive Care Med..

[10] Samransamruajkit R., Limprayoon K., Lertbunrian R., Uppala R., Samathakanee C., Jetanachai P., Thamsiri N. (2018). The Utilization of the Surviving Sepsis Campaign Care Bundles in the Treatment of Pediatric Patients with Severe Sepsis or Septic Shock in a Resource-Limited Environment: A Prospective Multicenter Trial. Indian J. Crit. Care Med..

[11] Balamuth F., Weiss S.L., Fitzgerald J.C., Hayes K., Centkowski S., Chilutti M., Grundmeier R.W., Lavelle J., Alpern E.R. (2016). Protocolized Treatment Is Associated with Decreased Organ Dysfunction in Pediatric Severe Sepsis. Pediatr. Crit. Care Med..

[12] Limprayoon K., Phumeetham S., Saito N. (2017). Effect of the ‘Surviving Sepsis Campaign 2012’ on Mortality in the Pediatric Department of Siriraj Hospital. S. Asian J. Trop. Med. Public Health.

[13] De Souza D., Machado F. (2019). Epidemiology of Pediatric Septic Shock. J. Pediatr. Intensive Care.

[14] Rudd K.E., Delaney A., Finfer S. (2017). Counting Sepsis, an Imprecise but Improving Science. JAMA.

[15] Han Y.Y., Carcillo J.A., Dragotta M.A., Bills D.M., Watson R.S., Westerman M.E., Orr R.A. (2003). Early reversal of pediatric-neonatal septic shock by community physicians is associated with improved outcome. Pediatrics.

[16] Khan M.R., Maheshwari P.K., Masood K., Qamar F.N., Haque A.U. (2012). Epidemiology and outcome of sepsis in a tertiary care PICU of Pakistan. Indian J. Pediatr..

[17] Wolfler A., Silvani P., Musicco M., Antonelli M., Salvo I., Italian Pediatric Sepsis Study (SISPe) Group (2008). Incidence of and mortality due to sepsis, severe sepsis and septic shock in Italian Pediatric Intensive Care Units: A prospective national survey. Intensive Care Med..

[18] Despond O., Proulx F., Carcillo J.A., Lacroix J. (2001). Pediatric sepsis and multiple organ dysfunction syndrome. Curr. Opin. Pediatr..

[19] Rusmawatiningtyas D., Rahmawati A., Makrufardi F., Mardhiah N., Murni I.K., Uiterwaal C.S.P.M., Savitri A.I., Kumara I.F., Nurnaningsih (2021). Factors associated with mortality of pediatric sepsis patients at the pediatric intensive care unit in a low-resource setting. BMC Pediatr..

[20] Davis A.L., Carcillo J.A., Aneja R.K., Deymann A.J., Lin J.C., Nguyen T.C., Okhuysen-Cawley R.S., Relvas M.S.,  Rozenfeld R.A., Skippen P.W. (2017). American College of Critical Care Medicine Clinical Practice Parameters for Hemodynamic Support of Pediatric and Neonatal Septic Shock. Crit. Care Med..

[21] Samransamruajkit R., Saelim K., Uppala R., Chaiyakulsil C., Suetrong B., Kongkiattikul L., Trepatchayakorn S., Law S., Itdhi-amornkulchai S., Aksilp C. (2024). A Thai guideline summary in the management of pediatric septic shock. Clin. Crit. Care.

[22] Carcillo J.A., Davis A.L., Zaritsky A. (1991). Role of early fluid resuscitation in pediatric septic shock. JAMA.

[23] Zhao X., Koutroulis I., Cohen J., Berkowitz D. (2019). Pediatric urgent care education: A survey-based needs assessment. BMC Health Serv. Res..

[24] Ventura A.M.C., Shieh H.H., Bousso A., Góes P.F., Fernandes I.d.C.F.O., de Souza D.C., Paulo R.L.P., Chagas F., Gilio A.E. (2015). Double-Blind Prospective Randomized Controlled Trial of Dopamine Versus Epinephrine as First-Line Vasoactive Drugs in Pediatric Septic Shock. Crit. Care Med..

[25] Rhodes A., Evans L.E., Alhazzani W., Levy M.M., Antonelli M., Ferrer R., Kumar A., Sevransky J.E., Sprung C.L., Nunnally M.E. (2017). Surviving Sepsis Campaign: International Guidelines for Management of Sepsis and Septic Shock: 2016. Intensive Care Med..

[26] Choudhary R., Watakulsin P., Promduangsi P., Chuenchom N., Khemla S., Lurchachaiwong W., Mock P., Heffelfinger J.D., MacArthur J.R., Bloss E. (2024). Underdiagnosis in clinical documentation of community-acquired sepsis among children admitted to hospitals in two rural provinces: Thailand, October–December 2017. BMJ Paediatr. Open.

[27] Miura S., Michihata N., Hashimoto Y., Matsui H., Fushimi K., Yasunaga H. (2023). Descriptive statistics and risk factor analysis of children with community-acquired septic shock. J. Intensive Care.

[28] Doymaz S., Rizvi M., Orsi M., Giambruno C. (2019). How Prepared Are Pediatric Residents for Pediatric Emergencies: Is Pediatric Advanced Life Support Certification Every 2 Years Adequate?. Glob. Pediatr. Health.

