# Supplementary material for: Survey of Thai Physicians’ Practice in Pediatric Septic Shock
Source: Children (Basel). 2024 May 15;11(5):597. doi: 10.3390/children11050597 (PMC11120040; doi:10.3390/children11050597)
Supplement: Supplementary file 1 [file children-11-00597-s001.zip › File S1.pdf]

## Supplement material S1

# Participant Questionnaire: Survey on the Management of Pediatric Sepsis and Septic Shock among Thai Physicians

### Part 1: General Information

- Gender: ☐ Male ☐ Female ☐ Prefer not to disclose ☐ Other: ____

- Years of Experience as a Physician

  ☐ 0-1 years ☐ 1-2 years ☐ 2-3 years

  ☐ 3-5 years ☐ 5-10 years ☐ More than 10 years

  ☐ Prefer not to disclose

- Medical specialist

  ☐ General Practice ☐ Pediatrics

  ☐ Prefer not to disclose ☐ Other: ____

- Current Professional Role

  ☐ Intern or General Practitioner (GP) ☐ Resident or clinical fellow

  ☐ Pediatric Specialty Physician or Faculty Staff

  ☐ Prefer not to disclose ☐ Other: ____

- Hospital Type Where You Work

  ☐ Primary care center (≤30 beds)

  ☐ Secondary care center (>30 beds)

  ☐ Tertiary care center

  ☐ Super tertiary care center

  ☐ Private Hospital

  ☐ Other: ____

- Geographical Region of Your Hospital

☐ Bangkok ☐ Central Region ☐ Northern Region

☐ Southern Region ☐ Eastern Region ☐ Northeastern Region

☐ Western Region

- Have you participated in any training or studied about the management of sepsis/septic shock in children?

☐ Yes ☐ No

**Part 2: Survey on Practices for Treating Pediatric Sepsis and Septic Shock**

**Please answer the survey truthfully.**

1. **When was the last time you treated a child with sepsis or septic shock?**

   ☐ Within the past week ☐ Within the past month

   ☐ Within the past 3 months ☐ Within the past 6 months

   ☐ Within the past year ☐ More than a year ago

   ☐ Never treated a child with sepsis or septic shock

2. **On average, how many patients did you diagnose with sepsis or septic shock per month in the past year?**

   ☐ 1-2 patients per month ☐ 2-5 patients per month

   ☐ 5-10 patients per month ☐ 10-15 patients per month

   ☐ More than 15 patients per month

3. **For a 6-year-old boy weighing 20 kg with no prior illnesses who was injured playing with friends in the park two days ago and now has a pus-filled wound on his leg,**

how long did it take to screen this patient until the doctor examined him at your hospital?

☐ 5 minutes ☐ 15 minutes ☐ 30 minutes

☐ 1 hour ☐ 2 hours ☐ Other: ____

4. Upon examining the patient described in question 3, a boy shows good consciousness, clear lungs, a wound with pus on the left leg, warm extremities, flash capillary refill, and bounding pulses, what do you consider the most important in the initial resuscitation of this patient?

☐ Broad-spectrum antibiotic ☐ Appropriate fluid bolus

☐ Wound dressing and sending ☐ pus for culture

☐ Dopamine infusion ☐ Epinephrine infusion

5. How soon after admission does a patient like the one in question 3 receive a broad-spectrum empirical antibiotic in your hospital?

☐ 15 minutes ☐ 30 minutes ☐ 1 hour

☐ 2 hours ☐ More than 2 hours ☐ Other: ____

6. For the patient in question 3, which fluid for resuscitation would you choose first?

☐ 5% Dextrose in saline ☐ Normal saline ☐ Ringer's lactate solution

☐ 5% human Albumin ☐ Blood component ☐ Other: ____

7. What volume of fluid would you use for loading fluid resuscitation for the patient in question 3?

☐ 200 ml (10 ml/kg) ☐ 300 ml (15 ml/kg) ☐ 400 ml (20 ml/kg)

☐ 500 ml (25 ml/kg) ☐ 600 ml (30 ml/kg) ☐ Other: ____

8. How would you administer the fluid in question 7 to the patient?

☐ IV bolus ☐ IV in 5 min ☐ IV in 10 min

☐ IV in 15 min ☐ IV in 30 min ☐ IV in 1 hr

☐ IV in 2 hr ☐ IV in 8 hr ☐ IV in 24 hr

☐ Per oral ☐ Intraosceous

☐ Fluid not required for this patient

9. **After the first fluid administration for the patient in question 3, if the BP is measured again at 75/40 mmHg and HR at 156 bpm with no signs of fluid overload, what is the maximum initial fluid resuscitation volume you would use before deciding to administer inotropic/vasoactive agents?**

☐ 400 ml (20 ml/kg) ☐ 500 ml (25 ml/kg) ☐ 600 ml (30 ml/kg)

☐ 800 ml (40 ml/kg) ☐ 1000 ml (50 ml/kg) ☐ 1200 ml (60 ml/kg)

☐ 1400 ml (70 ml/kg) ☐ 1600 ml (80 ml/kg) ☐ 1800 ml (90 ml/kg)

☐ 2000 ml (100 ml/kg) ☐ Other: ____

10. **Which inotropic/vasoactive agents are available for use in your hospital? (Multiple answers possible)**

☐ Dopamine ☐ Dobutamine ☐ Epinephrine

☐ Norepinephrine ☐ Milrinone ☐ Levosimendan

☐ Vasopressin ☐ Terlipressin ☐ Angiotensin

☐ Enoximone ☐ Other: ____

11. **After administering the full volume of fluid in question 9, how soon after you order treatment does the patient typically receive inotropic/vasoactive agents in your hospital?**

☐ 5 minutes ☐ 15 minutes ☐ 30 minutes

☐ 1 hour ☐ 2 hours ☐ Other: ____

12. If a child patient has a BP of 70/30 mmHg and a HR of 160 bpm with warm extremities after initial fluid administration, which inotropic/vasoactive agent would you choose first?

☐ Dopamine ☐ Dobutamine ☐ Epinephrine

☐ Norepinephrine ☐ Milrinone ☐ Other: ____

13. If a child patient has a BP of 60/40 mmHg and a HR of 160 bpm with cold extremities after initial fluid administration, which inotropic/vasoactive agent would you choose first?

☐ Dopamine ☐ Dobutamine ☐ Epinephrine

☐ Norepinephrine ☐ Milrinone ☐ Other: ____

14. If a child patient has been stabilized initially with fluids and epinephrine at 0.3 mcg/kg/min and is now in the ICU with BP 112/65 mmHg, HR 130 bpm, cold extremities, and ScvO2 < 70%, which inotropic/vasoactive agent would be most appropriate in this situation?

☐ Dopamine ☐ Dobutamine ☐ Epinephrine

☐ Norepinephrine ☐ Milrinone ☐ Levosimendan

☐ Vasopressin ☐ Terlipressin ☐ Angiotensin

☐ Enoximone ☐ Other: ____

15. During the treatment of a patient with sepsis/septic shock who has a urinary catheter placed, if the patient is not urinating, how long will you wait before considering referral to a pediatric nephrologist or deciding on further action?

☐ 1 hour ☐ 2 hours ☐ 3 hours

☐ 4 hours ☐ 5 hours ☐ 6 hours

☐ 7 hours ☐ 8 hours ☐ Other: ____

**16. What is the mortality rate for pediatric patients with sepsis/septic shock in your hospital?**

☐ Less than 10% ☐ 10-20% ☐ 20-40%

☐ 40-60% ☐ 60-80% ☐ More than 80%

☐ Other: ____

**17. Have you received training on pediatric sepsis/septic shock management during your training?**

☐ Yes ☐ No ☐ Other: ____

**18. Do you think there should be annual training on pediatric sepsis/septic shock management?**

☐ Yes, it should be organized

☐ No, it should not be organized

☐ Other: ____

**19. Are you able to access a central line in children (excluding umbilical vein catheters in newborns)?**

☐ Yes ☐ No ☐ Other: ____

**20. Who do you think should be able to access a central line in children? (Multiple answers possible)**

☐ General Practitioners ☐ Family Medicine Physicians

☐ Emergency Medicine Physicians ☐ General Pediatricians

☐ Pediatric Specialists ☐ General Surgeons

☐ Pediatric Surgeons ☐ Anesthesiologists

☐ Other: ____

21. Comments or suggestions on managing pediatric sepsis/septic shock in your hospital

................................................................................................................................................................................

22. Other comments or suggestions:

................................................................................................................................................................................
